# Autophagy is a promising process for linking inflammation and redox homeostasis in Down syndrome

**DOI:** 10.3389/fphar.2024.1491563

**Published:** 2024-10-02

**Authors:** Xuehai Ma, Weimin Li, Jun Ma, Zhongcheng Han, Shoulong Deng, Sutian Wang

**Affiliations:** ^1^ Xinjiang Key Laboratory of Mental Development and Learning Science, College of Psychology, Xinjiang Normal University, Urumqi, Xinjiang, China; ^2^ College of Physical Education and Health, East China Normal University, Shanghai, China; ^3^ Institute of Physical Education, Xinjiang Normal University, Urumqi, China; ^4^ Xinjiang Urumqi Youai Hospital, Urumqi, Xinjiang, China; ^5^ People’s Hospital of Xinjiang Uygur Autonomous Region, Urumqi, China; ^6^ Institute of Laboratory Animal Sciences, Chinese Academy of Medical Sciences and Comparative Medicine Center, Peking Union Medical College, Beijing, China; ^7^ State Key Laboratory of Swine and Poultry Breeding Industry, Guangdong Key Laboratory of Animal Breeding and Nutrition, Institute of Animal Science, Guangdong Academy of Agricultural Sciences, Guangzhou, China

**Keywords:** Down syndrome, autophagy, inflammation, redox homeostasis, stress signalling

## Abstract

Trisomy 21, characterized by the presence of an additional chromosome 21, leads to a set of clinical features commonly referred to as Down syndrome (DS). The pathological phenotypes observed in DS are caused by a combination of factors, such as mitochondrial dysfunction, neuroinflammation, oxidative stress, disrupted metabolic patterns, and changes in protein homeostasis and signal transduction, and these factors collectively induce neurological alterations. In DS, the triplication of chromosome 21 and the micronuclei arising from the missegregation of chromosomes are closely associated with inflammation and the development of redox imbalance. Autophagy, an essential biological process that affects cellular homeostasis, is a powerful tool to facilitate the degradation of redundant or dysfunctional cytoplasmic components, thereby enabling the recycling of their constituents. Targeting the autophagy process has been suggested as a promising method to balance intracellular inflammation and oxidative stress and improve mitochondrial dysfunction. In this review, we summarize the role of autophagy in regulating inflammation and redox homeostasis in DS and discuss their crosslinks. A comprehensive elucidation of the roles of autophagy in DS offers novel insights for the development of therapeutic strategies aimed at aneuploidy-associated diseases.

## 1 Introduction

Most people have two chromosomes 21, while those with three have Down syndrome (DS), also known as “trisomy 21”. DS is a complex disease that accumulates in many organs of the body, especially the skeletal musculoskeletal system, the nervous system, and the cardiovascular system, and affects about 1 in 800 newborns worldwide. Patients with DS are often characterized by short stature, hypotonia, decreased neuron numbers, cerebellar hypoplasia, intellectual disability, psychiatric disorders (such as depression and autism) and congenital heart defects (Do et al., 2017). In approximately 95% of DS cases, free trisomy 21 is observed, predominantly arising from errors during maternal meiosis I (around 66%) or meiosis II (approximately 21%), paternal meiosis I (about 3%) or meiosis II (5%), or post-zygotic mitotic errors (5%). Translocation is responsible for trisomy 21 in roughly 3.2% of cases. Mosaicism involving trisomy 21 is present in about 1.8% of individuals with DS. Partial trisomy 21 is uncommon and presents with a spectrum of symptoms that depend on the extent of the partial duplication of chromosome 21 (Antonarakis et al., 2020). Two primary hypotheses have been suggested to elucidate the biological dysfunction underlying the phenotypic traits of DS. The first hypothesis posits a specific gene-dosage effect from chromosome 21, including both the direct impact of overexpressed genes on HSA21 and the subsequent downstream effects. The second hypothesis involves developmental instability, where a nonspecific global alteration in gene expression due to the additional HSA21 leads to a disturbance in overall physiological homeostasis (Pritchard and Kola, 1999).

A variety of studies suggest people with DS show chronic immune dysregulation and mitochondrial dysfunction. On the one hand, individuals with DS exhibit higher incidences of various autoimmune disorders. At the molecular and cellular levels, those with trisomy 21 display evident markers of inflammation even without detectable infections (Espinosa, 2020). On the other hand, signs of mitochondrial dysfunction have been detected in cells and organs affected by trisomy 21. The mitochondrial characteristics in DS include diminished ATP production through oxidative phosphorylation, reduced respiratory capacity, impaired mitochondrial membrane potential generation, and structural changes in mitochondria (Izzo et al., 2018). Therefore, the inflammation, redox homeostasis and mitochondrial function in DS deserve more attention. Recently, some researchers found macroautophagy and selective autophagy play important roles in the course of DS and DS-related neurodegenerative disease and psychiatric disorders (including depression) (Bordi et al., 2019; Panagaki et al., 2024). Of note, autophagy plays a crucial role in coordinating inflammation, redox homeostasis and many disease processes. However, the interaction between them in DS remains unclear. Studying these issues will help improve the symptoms of DS. In this review, we outline the function of autophagy in DS. Additionally, we explore the potential interactions among autophagy, inflammation, oxidative stress, and redox balance, offering valuable insights for developing therapeutic strategies for DS.

## 2 Autophagy in Down syndrome

Autophagy, a fundamental intracellular metabolic mechanism, protects living organisms by breaking down and recycling excess or potentially harmful cytosolic components, thereby preventing toxic protein buildup and organelle dysfunction. Under normal conditions, autophagy maintains intracellular homeostasis. Impairment of autophagy is closely related to the pathogenesis of tumours, neurodegenerative diseases, metabolism-related diseases, and immune diseases. Lysosomal acidification defects and autophagy impairment were found in different stages of DS (Bordi et al., 2019; Tramutola et al., 2017). Numerous studies have shown that multiple pathways are involved in autophagy. Several of these pathways intersect at the target of rapamycin (TOR). It has been suggested that TOR is involved in cognitive decline, such as the progression of DS and AD (Perluigi et al., 2015). Troca-Marín JA’s research indicates that the mTOR signalling exhibits hyperactivity in the hippocampus of the Ts1Cje mouse model for DS (Troca-Marin et al., 2011). Moreover, some researchers believe the hyperactivation of the mTOR signalling in the DS brain leads to autophagy flux imbalances, which have negative effects on mitochondrial turnover (Iyer et al., 2014; Perluigi et al., 2014). A recent study conducted a comprehensive analysis of deficits in mitophagy and autophagy within fibroblasts from individuals with DS. Transmission electron microscopy analysis demonstrated that fibroblasts from individuals with DS contain a significantly higher number of mitochondria with abnormal morphology compared to diploid (2N) controls (Bordi et al., 2019). Further study revealed this phenotype is attributed to the impaired mitophagy responses in DS which are associated with PINK1/PARKIN dysregulation. In line with these findings, immunoblot analysis showed increased phosphorylation at S2448 within the kinase catalytic domain of mTOR in DS fibroblasts, suggesting abnormally heightened mTOR activity. Moreover, the elevated phosphorylation of ULK1 at S758 in DS fibroblasts indicates inhibited activity and reduced induction of autophagy. Inhibiting mTORC1 activity with AZD8055 rescues mitophagy deficiencies and alleviates macroautophagy suppression in DS fibroblasts. In addition, AZD also inhibits mTORC2, which in turn negatively regulates AKT and activates FOXO1 and FOXO3. These factors are crucial in controlling the expression of autophagy-related genes (Milan et al., 2015). A number of other studies have concluded that the activation of mTOR signalling in the frontal cortex from DS autopsy cases contributes to the production of fibrillar β-amyloid (Aβ) and the development of neurofibrillary tangles (NFT) (Perluigi et al., 2014). Actually, the mTOR hyperactivation in the DS hippocampus appears early and persists throughout postnatal development (Iyer et al., 2014). Hyperactivation of mTOR in these phases implies that autophagy is strongly inhibited.

**FIGURE 1:**
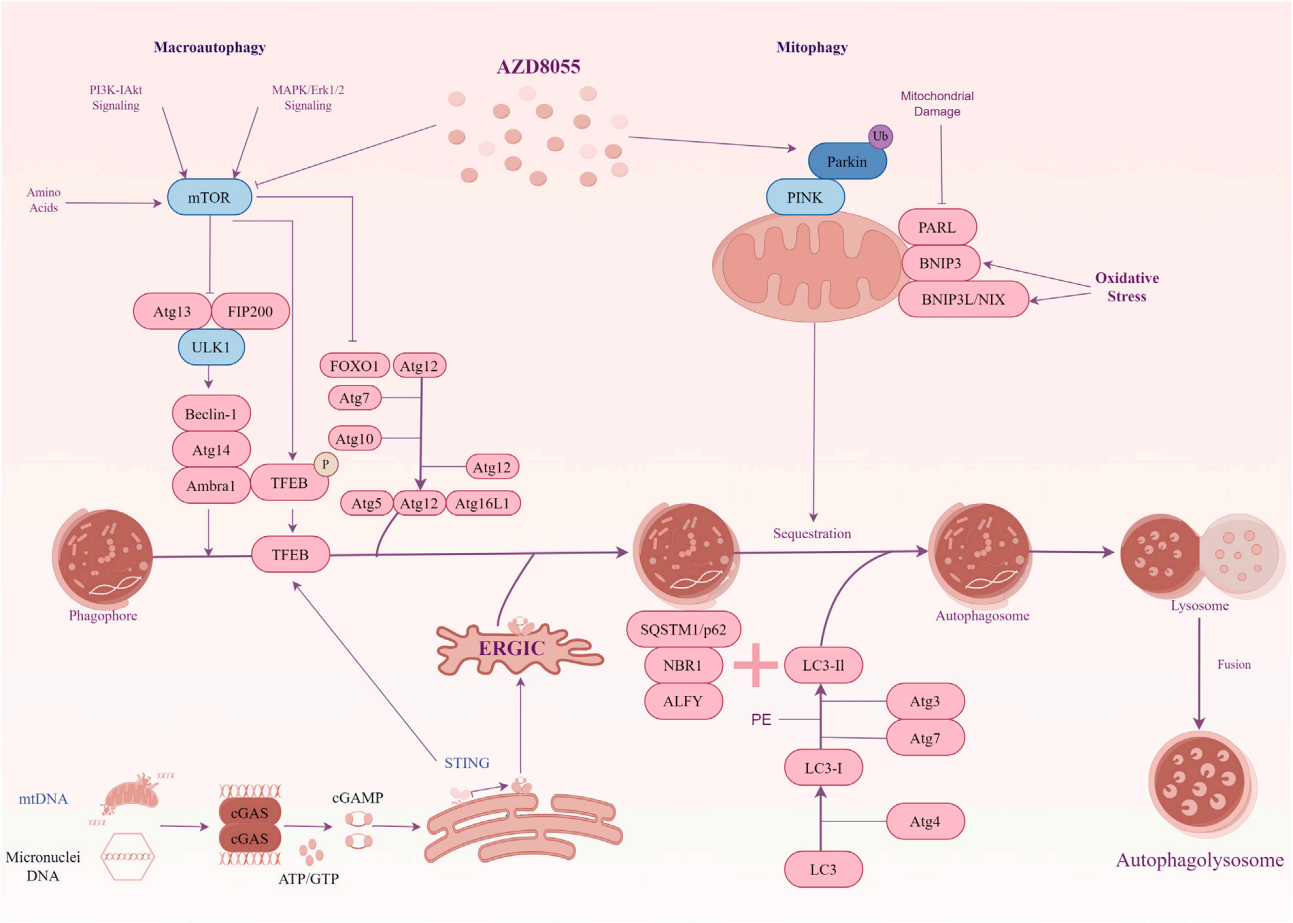
A diagrammatic representation illustrating the signal transduction of autophagy in Down syndrome. mTOR signalling exhibits hyperactivity in the hippocampus of the Ts1Cje mouse model for DS. The hyperactivation of mTOR results in the suppression of autophagy induction, and key proteins essential for autophagosome formation, such as ATG7 and FOXO1, are down-regulated. Inhibiting mTOR signalling restores mitophagy and autophagy flux, thereby facilitating the removal of damaged mitochondria. In addition, the cGAS-STING pathway participates in TFEB-mediated activation of autophagy in trisomy 21 cells. Genotoxic stress-induced DNA damage in trisomic cells results in the accumulation of double-stranded DNA in the cytoplasm, where the cytosolic receptor cGAS recognizes it. Upon activation, cGAS generates the signalling molecule cGAMP, which induces STING clustering and TBK1 activation, leading to IRF3-dependent transcription of target genes. Furthermore, activated STING promotes the translocation of TFEB to the nucleus independently of mTOR. TFEB stimulates the expression of lysosomal and autophagy-related factors, thereby enhancing autophagy and lysosomal degradation.

The increased number of damaged mitochondria and the micronuclei arising from the missegregation of chromosomes combine to cause an increase in cytoplasmic DNA levels in DS cells (also including other trisomic cells) (Krivega et al., 2021; Tiano and Busciglio, 2011). We are interested in the mechanisms by which organisms recognize cytoplasmic DNA and remove excess DNA from the cytoplasm in DS. CGAS-STING is a cytoplasmic DNA sensor that initiates inflammation and induces autophagy (Zhang et al., 2024). Initially, it was widely believed that the primary function of cGAS-STING was to mediate innate immunity. However, Chen et al. revealed autophagy induction via STING trafficking is a primordial function of the cGAS signalling (Gui et al., 2019). Given this function, cGAS-STING may be the key point linking intracellular DNA recognition and autophagic degradation in DS. In trisomic cells, an interesting study showed cytoplasmic dsDNA activated the cGAS-STING signalling (Krivega et al., 2021). This activation leads to the nuclear accumulation of the transcription factor IRF3, which subsequently initiates the type I interferon (IFN) response. Moreover, as a transcription factor that activates autophagy-related genes, the constitutive nuclear localization of TFEB is characteristic of human trisomic cells. Immunoblot analysis showed the autophagy marker genes were upregulated in these trisomic cells. These results indicate autophagy and lysosomal degradation are activated in cells with extra chromosomes. Further studies have confirmed the cGAS-STING signalling pathway triggers autophagy and TFEB-dependent transcription in these cell lines. Notably, the constitutive nuclear localization of TFEB occurs independently of mTOR in trisomic cells. An analysis of the activity of AKT1 and AMPK, known regulators of mTORC1, showed no consistent changes in p-AKT1-S473 and unchanged levels of p-AMPK-T172 in aneuploid cells compared to their diploid parental cells. Apparently, TFEB localization and autophagic flux are influenced by other cellular stresses. After eliminating cGAS and STING by CRISPR/Cas9, the absence of cGAS and STING significantly decreased the nuclear accumulation of TFEB, whereas this effect was not observed in diploid cells. It means the TFEB-dependent transcription in trisomic cells depends on the cGAS-STING signaling. However, the precise mechanism by which TFEB is regulated following the activation of the cGAS-STING pathway in trisomic cells remains unclear. The cGAS-STING not only triggers the IFN response but also initiates a transcriptional program that enhances autophagy and lysosomal biogenesis independently of mTORC1 signalling. These signals link the genetic instability of trisomic cells to the activation of autophagy (Figure 1).

## 3 Crosstalk between autophagy, inflammation and redox homeostasis in Down syndrome

### 3.1 Inflammation and autophagy in Down syndrome

In many co-morbidities associated with DS, the widespread and chronic immune dysregulation has received increasing attention. Studies have shown that immune cells in patients with DS exhibit an overactive inflammatory response, and levels of inflammatory factors in serum significantly increased, which contributes to an elevated susceptibility to infections, worse clinical outcomes, and chronic inflammation in this vulnerable population (Huggard et al., 2020). Several possibilities lead to abnormal inflammation in the body of a person with DS. Firstly, the chronic autoinflammation in people with DS is partly attributed to interferon hyperactivity, which is widely recognized to influence the inflammatory response. Four of the six interferon receptors are located on chromosome 21. On the one hand, individuals with trisomy 21 exhibit overexpression of IFN receptors encoded on chromosome 21 across various cell types. This increased expression is present not only at the mRNA level in both immune and non-immune cells but also on the cell surface (Sullivan et al., 2016). Secondly, both immune and non-immune cells in people with DS display hypersensitivity to IFN stimulation, as indicated by the enhanced induction of downstream JAK/STAT signalling and IFN-stimulated genes (Waugh et al., 2019). Moreover, a variety of studies have identified alterations suggestive of chronic autoinflammation in individuals with DS. These changes include elevated levels of various cytokines and chemokines that are known to function downstream of IFN signalling (Espinosa, 2020). Thus, when other pathogenic microorganisms invade, individuals with DS are more likely to experience a more intense and prolonged cytokine storm. In addition to IFN, NF-κB is a key regulator of the inflammatory response. Studies have shown that the activity of the NF-κB signalling is significantly enhanced in patients with DS, which may be an important cause of the chronic inflammatory state (Engidawork et al., 2001). Activation of NF-κB can exacerbate the inflammatory response by promoting the production of inflammatory factors, such as IL-6, TNF-α, and IL-1β, through multiple pathways (Barnabei et al., 2021). Furthermore, mitochondrial function is significantly impaired in individuals with DS, which leads to increased oxidative stress and ROS production (Tan et al., 2023). These ROS can activate the inflammatory response through multiple pathways, including activation of NF-κB and NLRP3 inflammasome. Inflammasome activation leads to an inflammatory response involving the cleavage of pro-IL-1β and IL-18 into active forms. NLRP3 inflammasomes have been well-studied and have been associated with a wide range of diseases. Studies have shown that children with DS have significantly elevated levels of IL-1β and are more susceptible to autoimmune diseases (Huggard et al., 2020). Therefore, inflammasomes and their potential immunomodulatory role are potential targets for further research in DS.

### 3.2 The interaction between autophagy and redox homeostasis in Down syndrome

In addition to inflammation, the neuropathology of DS includes altered redox homeostasis as well as mitochondrial dysfunction. Oxidative stress is involved in both processes. Individuals with DS usually exhibit a significant state of oxidative stress. This state is due to increased levels of ROS and RNS in the body as well as weakened antioxidant defence mechanisms. Many studies have confirmed that serum levels of ROS and RNS are significantly elevated in patients with DS (Busciglio and Yankner, 1995; Zis et al., 2012). Meanwhile, antioxidant defence mechanisms are significantly weakened in patients with DS. It is manifested by reduced activity of antioxidant enzymes and reduced levels of antioxidants. For example, the activities of CAT and GPX are significantly reduced in patients with DS (Valenti et al., 2018). Moreover, serum levels of vitamin C, vitamin E, and GSH are significantly lower in patients with DS (Parisotto et al., 2015).

The molecular mechanisms that cause redox imbalance in DS have been the focus of attention. Firstly, the triplication of various Hsa21 genes, including SOD1, APP, and BACH1, are thought to contribute to disrupting redox homeostasis in individuals with DS or in animal models. Superoxide dismutase 1 (SOD1) is a powerful endogenous enzyme relevant to neural function, which binds copper and zinc and is encoded on chromosome 21. The overexpression of SOD1, without the presence of a peroxide-detoxifying enzyme in the same cellular compartment, leads to increased oxidative stress. In various DS cells and tissues, SOD1 levels were discovered to be approximately 50% higher than normal (de Haan et al., 1995). The triplication of the APP gene (about 50% higher protein level in DS mouse models) is another significant factor supporting the oxidative stress hypothesis of neurodegeneration in DS (Chen et al., 2021). In individuals with DS, increased expression of the APP gene results in heightened production and deposition of Aβ, which is associated with the production of ROS (Barone et al., 2018). In turn, the overproduction of ROS induced by trisomy 21 may alter APP processing, leading to the intracellular accumulation of Aβ. BACH1, which is encoded on chromosome 21, plays a role in regulating the antioxidant response in DS. A study showed that the protein level of BACH1 in Ts65Dn mice (DS animal models) is 50% higher than that of 2N individuals (Di Domenico et al., 2015). BACH1 can attach to the antioxidant response elements in DNA, thereby inhibiting the activation of glutamate-cysteine ligase, GST, HO-1 and other antioxidant proteins (Barone et al., 2018). Mitochondrial dysfunction is another important cause of redox imbalance in DS. Studies have shown that mitochondrial function is significantly impaired in patients with DS, leading to increased ROS production and decreased ATP production. Mitochondrial dysfunction not only leads to increased oxidative stress but also affects cellular energy metabolism and signalling, which can exacerbate disease progression (Ganguly and Kadam, 2023). Furthermore, there is a complex interaction between oxidative stress and inflammation. Oxidative stress can activate the inflammatory response through a variety of pathways, for example, through activation of NF-κB and NLRP3 inflammasomes (Cao et al., 2023). In turn, the inflammatory response can exacerbate oxidative stress by generating ROS and RNS, thus creating a vicious cycle. NF-κB is a key regulator of oxidative stress and inflammatory responses. Studies have shown that the activity of the NF-κB signalling pathway is significantly increased in patients with DS, which may be an important contributor to the chronic inflammatory state and oxidative stress (Engidawork et al., 2001). The activation of NF-κB can promote the production of inflammatory factors, such as IL-6, TNF-α, and IL-1β, through multiple pathways, thereby exacerbating inflammatory responses and oxidative stress (Morgan and Liu, 2011). Nrf2 is another key regulator of the antioxidant response. Studies have shown that the activity of the Nrf2 is significantly reduced in patients with DS, which may be an important factor contributing to the attenuation of antioxidant defence mechanisms (Pagnotta et al., 2022). The activation of Nrf2 can alleviate oxidative stress by promoting the expression of antioxidant enzymes and antioxidants.

### 3.3 Linking redox homeostasis and inflammation through autophagy in Down syndrome: therapeutic targets or partners?

Studies have confirmed that the autophagy process is closely linked to inflammatory responses, especially in some neurodegenerative diseases (Bostanciklioglu, 2019). There is no doubt that the interaction between autophagy and inflammatory response plays an important role in the course of DS. Here, we inferred the possible molecular mechanisms and pathways underlying the relationship between autophagy and inflammation interactions in DS. Autophagy can reduce inflammatory responses by degrading inflammatory signalling molecules and damaged mitochondria. For example, autophagy can reduce inflammation by removing damaged mitochondria and reducing ROS production (Mizushima and Levine, 2020). Moreover, autophagy can inhibit the inflammatory response by degrading the NLRP3 inflammasome (Harris et al., 2011). In turn, inflammatory signalling can affect the autophagy process through multiple pathways. Of these, TNF-α and IL-1β can inhibit autophagy by activating the NF-κB signalling (Levine et al., 2011). In addition, oxidative stress generated in chronic inflammatory states can also inhibit autophagy (Zhang et al., 2016). Moreover, studies have shown that activation of the cGAS-STING signalling not only induces the production of IFN and other inflammatory cytokines but also promotes autophagy and lysosomal biosynthesis through multiple pathways (such as TFEB signalling) in DS or trisomic cells (Sullivan et al., 2017). The PI3K-AKT-mTOR axis also plays an important role in autophagy-inflammation interactions. The PI3K-AKT-mTOR pathway is always overactivated in DS patients. Studies have shown that activation/inhibition of the PI3K-AKT-mTOR pathway can exacerbate/attenuate inflammatory responses by inhibiting autophagy (Gomez et al., 2020). Chronic inflammation, overproduction of inflammatory factors, and abnormal activation of inflammatory pathways are the main features of the inflammatory response in patients with DS. Autophagy plays an important role in regulating the inflammatory responses, and inflammatory signals also affect the autophagic process through multiple pathways. Understanding these mechanisms not only helps to unravel the pathological mechanisms of DS but also provide new targets for the treatment of the disease.

Autophagy plays an important role in regulating redox homeostasis. Macroautophagy and selective autophagy can reduce oxidative stress by degrading damaged mitochondria and harmful proteins to reduce ROS production (Mizushima and Levine, 2020). In addition, macroautophagy can inhibit inflammatory responses by degrading NLRP3 inflammasomes, thereby attenuating oxidative stress (Takahama et al., 2018). Similarly, oxidative stress can affect the autophagy process through multiple pathways. For example, ROS can promote autophagy by activating AMPK and inhibiting mTOR (Levine et al., 2011). However, excessive oxidative stress can also inhibit autophagy by damaging autophagy-related proteins (Bhatti et al., 2017). The PI3K-AKT-mTOR axis plays an important role in the interaction between autophagy and redox homeostasis. Studies have shown that activation of the PI3K-AKT-mTOR axis can exacerbate oxidative stress by inhibiting autophagy (Kim et al., 2018). Inhibition of the PI3K-AKT-mTOR axis can reduce oxidative stress by promoting autophagy (Levine et al., 2011). In DS fibroblasts, the downregulation of mTOR-dependent macroautophagy inhibits mitophagy activity. Deficient mitophagy cannot clear damaged mitochondria, which leads to increased oxidative stress (Bordi et al., 2019). Moreover, the heightened oxidative stress in DS cells, caused by reduced clearance of dysfunctional mitochondria, may further impair the autophagy-mitophagy process, establishing a negative regulatory feedback loop. The restoration of mitophagy and redox homeostasis with an mTOR inhibitor reveals common sites of regulation, providing a new target for ameliorating DS. We have previously outlined the function of cGAS-STING signaling to mediate autophagy. Recent studies have identified STING as an upstream regulator of cellular oxidative stress. Activation of the cGAS-STING pathway can modulate lipid peroxidation and ROS levels through the downstream signal ISG15. ISG15, a member of the ISG family, induces IFN expression, facilitates “protein ISGylation,” and interferes with ubiquitin modifications. Through ISG15, STING can negatively regulate the ubiquitin-proteasome system, leading to increased interferon-mediated ROS production (Zhang et al., 2024). Currently, more studies are needed to analyze the relationship between autophagy and redox homeostasis in DS.

## 4 Conclusion and prospects

**FIGURE 2:**
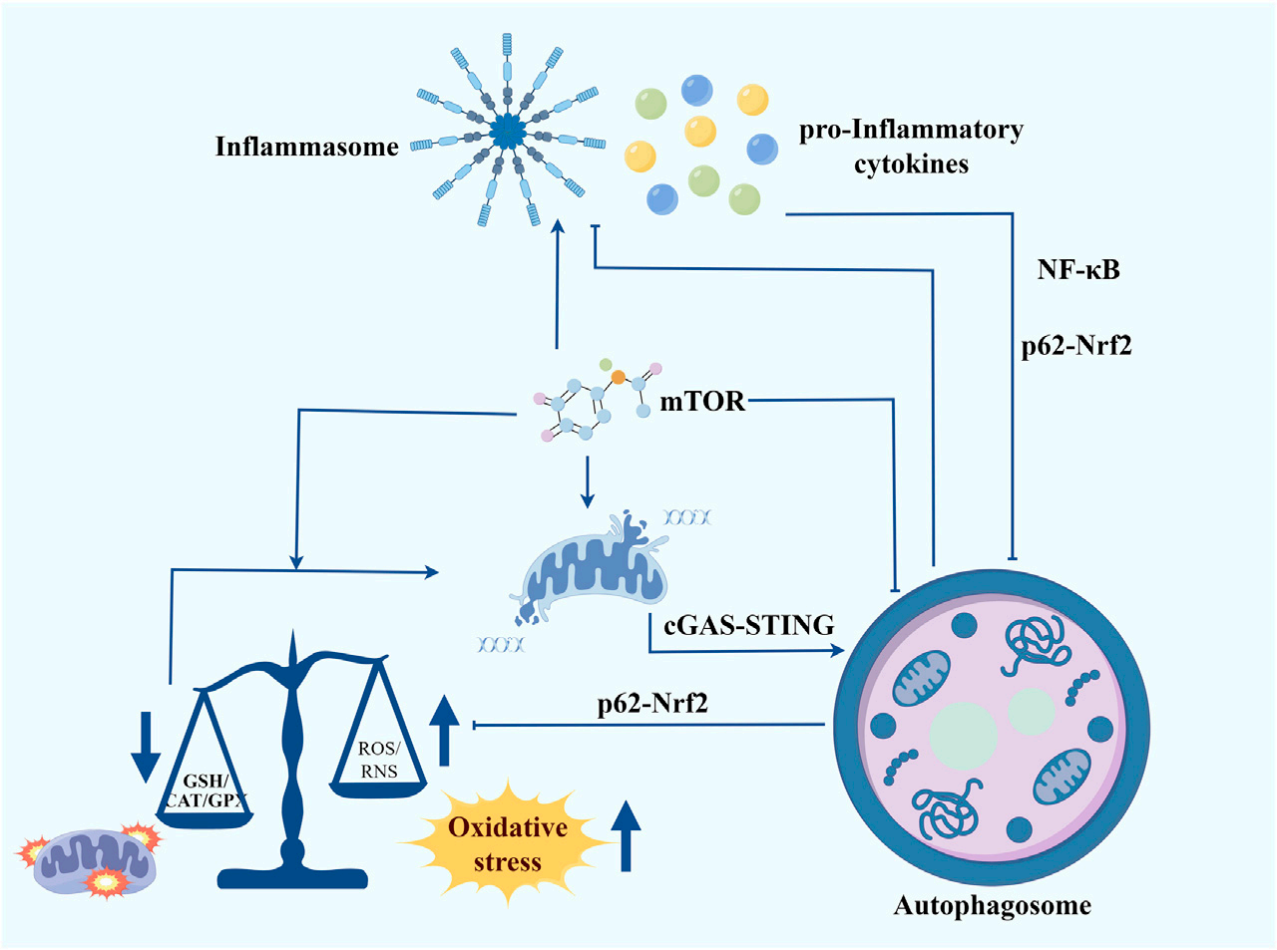
Autophagy contributes to regulating inflammation, maintaining redox homeostasis and improving mitochondrial function in Down syndrome. Inflammatory responses and oxidative stress are autophagy triggers. In turn, autophagy also participates in regulating the levels of pro-inflammatory cytokines and cellular redox homeostasis. mTOR serves as a crucial negative regulator of autophagy. The mTOR signalling pathway is significantly more active in the cells of individuals with DS, potentially leading to autophagy inhibition and heightened oxidative stress. Inhibiting the mTOR pathway can promote autophagy, thereby reducing inflammation and oxidative stress and improving mitochondrial function. Nrf2, a key regulator of the cellular antioxidant response, enhances antioxidant capacity by promoting the expression of antioxidant enzymes, thus mitigating oxidative stress. Additionally, Nrf2 influences inflammation and redox balance through p62-mediated autophagy. Another signalling pathway of interest is cGAS-STING. A notable study found that in trisomy 21 cells, the accumulation of cytoplasmic double-stranded DNA activated the cGAS-STING pathway. Given its ability to regulate autophagy, interferon signalling, and oxidative stress, cGAS-STING presents a promising target for alleviating DS symptoms.

The phenotypes associated with DS are quite complex. Therefore, we need to take a more integrated and multifaceted consideration of the intrinsic molecular mechanisms that trigger DS and try to find common mechanisms that lead to different disorders. Integrating the results of multiple studies, chronic inflammation and redox homeostasis imbalance may be the key points connecting most of the DS phenotypes (Figure 2). Meanwhile, autophagy is an important node connecting inflammation and redox homeostasis. On one hand, inflammatory responses and oxidative stress are autophagy triggers. On the other hand, autophagy also participates in regulating the levels of pro-inflammatory cytokines and cellular redox homeostasis. mTOR is a key negative regulator of autophagy. Studies have shown that the activity of the mTOR signalling pathway is significantly increased in the cells of patients with DS, which may be an important cause of autophagy inhibition and increased oxidative stress. By inhibiting the mTOR, autophagy can be promoted, thereby reducing inflammation and oxidative stress and ameliorating mitochondrial dysfunction. As a key regulator of cellular antioxidant response, Nrf2 can enhance cellular antioxidant capacity by promoting the expression of antioxidant enzymes, thereby attenuating oxidative stress. Meanwhile, Nrf2 can also regulate inflammation and redox balance by affecting p62-mediated autophagy (Jiang et al., 2015). Another signalling to focus our attention on is the cGAS-STING. An interesting study revealed that in trisomy 21 cells, the accumulation of cytoplasmic double-stranded DNA activated the cGAS-STING signalling pathway (Krivega et al., 2021). Given its combined ability to regulate autophagy, interferon signalling, and oxidative stress, we believe that cGAS-STING will be a promising target for alleviating the symptoms of DS. The next step should involve a more comprehensive analysis of the relationship between autophagy, inflammation, and redox homeostasis. Additionally, further evidence should be sought to clarify their mechanisms in DS.
